# Plasmapheresis for the management of acute cyanide poisoning: A case report and review of literature

**DOI:** 10.1002/ccr3.4228

**Published:** 2021-06-24

**Authors:** Navid Namakizadeh Esfahani, Shafeajafar Zoofaghari, Amirhossein Akhavan Sigari, Gholamali Dorooshi

**Affiliations:** ^1^ Isfahan University of Medical Sciences Isfahan Iran; ^2^ Isfahan Clinical Toxicology Research Center Department of Clinical Toxicology Khorshid Hospital Isfahan University of Medical Sciences Isfahan Iran

**Keywords:** cyanide toxicity, emergency medicine, plasmapheresis, toxicology

## Abstract

In case of mild to moderate cyanide poisoning, especially when standard antidote kits are not readily available, plasmapheresis can be utilized as an alternative option alongside supportive measures.

## Graphical abstract

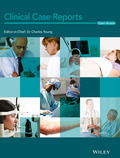

## 1 INTRODUCTION

Cyanide is a well‐known cause of intoxication and a historically recognized substance for suicide.^1^ Cyanide salts are widely used in the jewelry industry and are not readily available to the public. Thus, it is a common cause of intoxication among workers in the jewelry industry.^2^, ^3^ By disrupting the enzymatic pathways involved in cellular respiration, cyanide hinders the utilization of oxygen by cells, leading to adenosine triphosphate (ATP) depletion, and ultimately, cellular dysfunction. Cyanide‐induced hypoxia eventually leads to neurologic and cardiovascular compromise.^4^ Rapid diagnosis of cyanide poisoning may not be possible due to its nonspecific presentations, limited availability of blood cyanide level kits, and their time‐consuming nature. Patients with acute cyanide poisoning can present with headache, confusion, vertigo, palpitations, respiratory distress, and hyperventilation. Rapid development of hypotension, shock, bradycardia, and ultimately death may ensue.^4^ Cellular hypoxia leads to anaerobic cellular metabolism, accumulation of lactic acid, and eventually, lactic acidosis. Several studies have pointed to lactate levels as a sensitive marker in cyanide poisoning.^4^ Management of cyanide poisoning mainly consists of supportive measures in order to preserve organ perfusion and maintain proper homeostasis. Antidote kits are used in emergency settings and have proven efficacy in improving survival; however, these kits may not be available at all centers. Other measures such as hemodialysis and plasmapheresis may be used on a case by case basis to eliminate toxic substances from the circulation.^2^, ^5^, ^6^

In this report, we aim to discuss plasmapheresis as an acceptable alternative measure in patients with cyanide poisoning, and in centers where cyanide antidote kits are not available.

## 2 CASE PRESENTATION

A 39‐year‐old male patient was brought to the emergency department with severe nausea and vomiting after intentional ingestion of two spoonsful of cyanide salt which had been obtained from a friend in the jewelry industry. The patient was admitted to our center approximately 7 h after ingestion of the substance. Immediately after the ingestion of the powder, gastric irrigation and activated charcoal administration were undertaken in a primary health care facility, and the patient was then referred to our center due to the development of refractory nausea and severe vomiting.

Upon arrival, the patient had an oral temperature of 37.2°C, pulse rate of 110 beats per minute, respiratory rate of 18 breaths per minute, blood pressure recording of 110/70, and a Glasgow Coma Scale (GCS) score of 15/15. Pulse oximetry showed 97% oxygen saturation with a regular pulse. Peripheral perfusion was assessed using capillary refill time and was normal. Physical examination other than an evident tachycardia was otherwise normal. The patient reported tobacco use but no history of alcohol abuse or addiction to illicit drugs.

After admission to the emergency department, primary blood work revealed leukocytosis and metabolic acidosis (laboratory data are summarized in Table 1). An electrocardiogram (ECG) of the patient showed normal sinus rhythm and tachycardia.

**TABLE 1:** Laboratory data of the patient upon admission

Laboratory data	Value	Normal range
MCH	30.3	26‐32
MCHC	34.8	32‐36
MCV	87	80‐100
RDW	12.9	11‐13
WBC	14.2	4‐10
RBC	5.75	3.9‐5.9
Hemoglobin	17.4	14‐18
Hematocrit	50.0	42‐52
Platelet	149	150‐450
Lymphocytes	10.2%	
Neutrophils	86.4%	
Bun	16	8.8‐20.5
Cr	1.45	0.86‐1.4
AST	32	10‐40
ALT	24	10‐41
Na	139	136‐145
K	3.9	3.5‐5.1
BS	120	70‐135
PTT	35.2	28‐40
PT	11	10‐12
INR	1.1	1‐1.2
Ca	7.89	8.6‐10
Inorganic phosphate	3.47	2.6‐4.5
Alb	3.7	3.9‐4.9
MG	1.59	1.8‐2.6
ESR	3	0‐12

An intravenous line was obtained and hydration coupled with other supportive measures was started. After 2 h, the patient's blood pressure dropped to 67/35 and peripheral perfusion decreased. Based on the history obtained from the patient, and the metabolic acidosis along with hypotension, cyanide poisoning was highly suspected. Due to the unavailability of cyanide antidote kits at our center, management consisted of norepinephrine 10‐20 micrograms per minute (mcg/min) for blood pressure support, sodium bicarbonate for metabolic acidosis, and two sessions of plasmapheresis via a right femoral access using a hemodialysis set up with the appropriate filter. The duration of each plasmapheresis session was 3 h. In the first session, 3 L of plasma was replaced with 2300 mL of normal saline (N/S) and 700 mL fresh frozen plasma (FFP). In the second session, 3 L of plasma was replaced with 2200 mL of N/S, 700 mL of FFP, and two 50 cc vials of 20% albumin. The two plasmapheresis sessions were 5 h apart and the time from toxic ingestion to the first plasmapheresis was 10 h.

After the completion of two sessions of plasmapheresis, hypotension and acidosis resolved. Venous blood gas (VBG) measurements are summarized in Table 2. The patient recovered completely after 2 days and was discharged after psychological evaluation.

**TABLE 2:** Venous blood gas (VBG) results

Measures	Upon admission	After first plasmapheresis session	After second plasmapheresis session	Normal values
pH	7.30	7.45	7.42	7.32‐7.43
HCO_3_	12.6	26.1	26.7	23‐29
PCO_2_	26.3	37.4	41.4	41.51
PO_2_	63.7	49.3	54.1	30‐40
BE	‐11.8	2.4	2.0	
BB	37.1	51.2	52.3	

## 3 DISCUSSION

Early recognition of cyanide toxicity in the emergency department is of utmost importance. Patients with cyanide toxicity can present with a variety of manifestations based on the amount of substance consumed, the time past the consumption, and the route of exposure. In this paper, we have presented a case of a 39‐year‐old man with acute cyanide poisoning who was treated with plasmapheresis in conjunction with other supportive measures.^7^ Plasmapheresis eliminates toxins by removing plasma proteins and toxins present in the plasma through exchange of the patient's plasma with a suitable solution. The efficacy of plasmapheresis in toxin removal is related to several factors such as the molecular weight, volume of distribution, and protein binding of the toxin.^8^

Although cyanide exposure can occur unintentionally in occupational settings such as mining, agriculture, and metal industries, intentional ingestion of cyanide salts has been used for suicide and murder. Whereas the clinical presentations and laboratory data may not be specific at the time of presentation, some patients such as ours may confess to have intentionally ingested toxic cyanide salts.^3^

Patients with exposure to cyanide can present with altered levels of consciousness, seizures, respiratory compromise, nausea, vomiting, cardiac arrhythmias and arrest, blood pressure changes, altered body temperature, etc secondary to the cellular anoxia caused by cyanide. Organs with a high rate of oxygen consumption such as the brain, heart, and kidneys are prominently susceptible to damage. Although cherry red skin lesions and bitter almond odor of the breath may be more specific of cyanide toxicity, they are only present in a minority of patients (~15%).^7^, ^9^

Primary management of cyanide poisoning is similar to the management of other toxicities, supportive care. Oxygen supplementation, hydration, and removal of the toxic source are the mainstay of therapy. Patients' vital signs and cardiac function should be monitored continuously. Intubation and vasopressors are inevitable if respiratory compromise or refractory hypotension occur, respectively. When available, a cyanide antidote kit which consists of sodium thiosulfate, sodium nitrite, and amyl nitrite must be used. Amyl nitrite and sodium nitrite cause the dissociation of cyanide from the cellular cytochrome oxidase, leading to cellular relief and reversion to aerobic metabolism. The methemoglobinemia secondary to the use of nitrites will then be resolved by sodium thiosulfate. The end result is the urinary excretion of thiocyanate.^7^

In our case, apart from vigilant supportive management, due to the unavailability of the cyanide antidote kit, we utilized plasmapheresis for intoxication. Plasmapheresis is not a common measure in cases of cyanide poisoning. However, there have been sparse reports of plasmapheresis in such cases.

Z. Liu et al^2^ reported a case of a 24‐year‐old male patient who was diagnosed with concurrent methanol and cyanide poisoning. The patient had presented with bradycardia, hypotension, and decreased consciousness. Aside from supportive management, the patient was started on plasmapheresis 3 h of postingestion. The procedure was 2 h, during which 2400 mL of plasma was removed from the patient and replaced with 3000 mL of replacement solution consisting of 2000 mL FFP and 1000 ml N/S. The patient had undergone hemodialysis after plasmapheresis. In our case, the patient did not have any concurrent poisoning alongside cyanide ingestion and did not undergo hemodialysis.

In another case, Shatila et al^10^ introduced a case of a young individual who presented to the emergency department with low oxygen saturation and increased methemoglobinemia. Supportive measures were taken and the patient was started on plasmapheresis on the second day of admission, primarily due to a suspected thrombotic thrombocytopenic purpura (TTP). Plasmapheresis continued for 6 days after which methemoglobin levels were normalized. Although cyanide toxicity does not directly cause methemoglobinemia, it can be induced by utilizing nitrites in such cases. When only nitrates are available, induction of methemoglobinemia and subsequent plasmapheresis may be an option.

## 4 CONCLUSION

Cyanide poisoning can present with nonspecific symptoms which can complicate the course of diagnosis and therapy. The clinical course can vary from mild to severe. Early detection of cyanide toxicity can help guide the treatment and improve outcomes. In our case, due to the unavailability of cyanide antidote kits, we used plasmapheresis alongside other supportive measures. Treatment guidance based on a case report is not possible. However, plasmapheresis can be done when standard treatment is not available, the patient is not responding to such therapy, or concurrent intoxications exist.

## CONFLICT OF INTEREST

None declared.

## AUTHOR CONTRIBUTIONS

NNE, SZ, and GD: presented the case and were involved in the management of the patients and gathered relevant information from the case. AAS, NNE, and SZ: wrote the primary and final draft of the manuscript. The final article was read and approved by all authors for publication.

## ETHICAL APPROVAL

Patient signed the informed consent and accepted the publication of his data for the purpose of publication in a scientific journal. All procedures performed in this study were in accordance with the ethical standards of the institutional research committee and with the 1964 Helsinki declaration and its later amendments or comparable ethical standards.

## CONSENT TO PUBLISH

The patient's consent was taken for the publication of his data in a scientific journal.

## Data Availability

Data sharing is not applicable to this article as no datasets were generated or analyzed during the current study.
